# Phi Index: A New Metric to Test the Flush Early and Avoid the Rush Hypothesis

**DOI:** 10.1371/journal.pone.0113134

**Published:** 2014-11-18

**Authors:** Diogo S. M. Samia, Daniel T. Blumstein

**Affiliations:** 1 Departamento de Ecologia, Universidade Federal de Goiás, Goiânia, Goiás, Brazil; 2 Department of Ecology and Evolutionary Biology, University of California Los Angeles, Los Angeles, California, United States of America; Università degli Studi di Napoli Federico II, Italy

## Abstract

Optimal escape theory states that animals should counterbalance the costs and benefits of flight when escaping from a potential predator. However, in apparent contradiction with this well-established optimality model, birds and mammals generally initiate escape soon after beginning to monitor an approaching threat, a phenomena codified as the “Flush Early and Avoid the Rush” (FEAR) hypothesis. Typically, the FEAR hypothesis is tested using correlational statistics and is supported when there is a strong relationship between the distance at which an individual first responds behaviorally to an approaching predator (alert distance, AD), and its flight initiation distance (the distance at which it flees the approaching predator, FID). However, such correlational statistics are both inadequate to analyze relationships constrained by an envelope (such as that in the AD-FID relationship) and are sensitive to outliers with high leverage, which can lead one to erroneous conclusions. To overcome these statistical concerns we develop the phi index (Φ), a distribution-free metric to evaluate the goodness of fit of a 1∶1 relationship in a constraint envelope (the prediction of the FEAR hypothesis). Using both simulation and empirical data, we conclude that Φ is superior to traditional correlational analyses because it explicitly tests the FEAR prediction, is robust to outliers, and it controls for the disproportionate influence of observations from large predictor values (caused by the constrained envelope in AD-FID relationship). Importantly, by analyzing the empirical data we corroborate the strong effect that alertness has on flight as stated by the FEAR hypothesis.

## Introduction

Animals must escape predators because failure to do so can result in death and termination of any future contribution to fitness. However, escaping too early can also result in a loss of benefits such as finding food or a mate. Optimal escape theory predicts that prey flee from predators at the point in which risk and cost are equal [1], [2]. Because of the relative ease of studying flight initiation distance (FID, the predator-prey distance when escape begins), the theory has been widely supported by a number of studies since its publication. Importantly, while there is a species-specific signal to FID [3], within species, it is affected by many different variables that may include both internal factors, such as age, sex, condition, and pregnancy, as well as external factors, such as temperature, season, degree of human impact, distance to cover, and relative exposure to predators (e.g., [4]–[8]). Understanding the distance at which an individual flees an approaching predator is of more than mere academic interest because animals may view humans as predators [9], and FID to humans has been used to develop set-back zones to reduce human disturbance on wildlife [10]–[13].

While many factors are correlated with FID, a previous study [14] showed that most of the 63 Australian birds studied had a significant positive correlation between FID and starting distance, a proxy of alert distance (AD, the predator-prey distance when the prey becomes aware of and begins to monitor the predator; [15]–[17]). The positive relationship between AD and FID was also frequently reported in other taxa (e.g., [18], [19]).

The finding that prey seem to adjust flight according to the distance at which the predator was detected was difficult to explain with previously existing economic escape theory [20]. Thus, the ‘flush early and avoid the rush’ (FEAR) hypothesis was proposed and stated that animals will flee from an approaching predator soon after starts monitoring it in order to minimize costs incurred by monitoring predator behavior [21]. Ongoing monitoring is expected to increase costs by diverting attention away from beneficial activities, as well as by incurring energetic costs [22], although these latter costs may be modest compared to other costs (such as lost opportunity costs from flight, as well as the costs of not-fleeing; [20]). Nonetheless, a recent meta-analysis found substantial support consistent with the FEAR hypothesis in birds and mammals [23].

**Figure 1:**
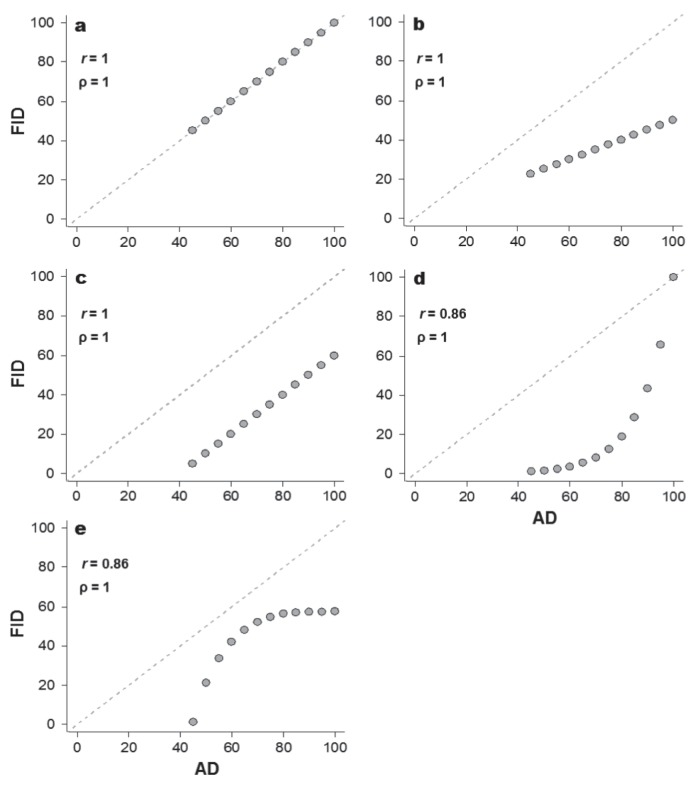
Examples of how a correlation coefficients (Pearson’s *r* and Spearman’s ρ) can lead to misleading conclusions about the flush early and avoid the rush (FEAR) hypothesis. In panel a, individuals flush immediately upon responding to a predator. Panels b and c illustrate other strategies that lead to identical correlation coefficients despite individuals not flushing immediately after response. Panels d and e illustrate how other non-linear monotonic relationships can result in ρ = 1. AD, alert distance; FID, flight initiation distance. The dashed line represents a 1∶1 relationship.

Most previous studies have used linear correlation analysis to assess the support for the FEAR hypothesis; a large positive correlation between AD and FID would suggest strong support (example in Fig. 1a). If prey flush as soon as they begin monitoring the predator, the FEAR hypothesis predicts a 1∶1 relationship between AD and FID (in terms of linear regression analysis, intercept = 0, slope = 1 and *r* = 1, Fig. 1a). However, it is important to note that large positive correlations (e.g., Pearson’s *r*∼1) do not necessarily mean that prey flush as soon as they begin monitoring the predator because other linear relationships between AD and FID are also theoretically possible. Hypothetical linear correlations between AD and FID under departure from the 1∶1 expectations are illustrated in Figures 1b and 1c. Figure 1b depicts a scenario in which prey always wait for an initial distance after it begins monitoring a predator and flushes at intermediate distances between immediate flight and not fleeing (i.e., FID∼½ AD, slope <1). Figure 1c illustrates a scenario in which prey systematically wait for predators to travel 40 m before initiating flight (intercept <0 m).

Additionally, the linear correlation analyses used to test the FEAR hypothesis are critically sensitive to outliers, which becomes even more important when sample sizes are relatively low [24]–[26]. Influential observations, defined as an observation which simultaneously lies near an extreme in the space of predictor and response variables [26], are especially troublesome. In extreme cases, an observation with a high influence could make a positive AD-FID relationship become negative.

The Spearman’s rank correlation (coefficient denoted by the Greek letter rho, ρ) could be used as a non-parametric alternative to test the FEAR hypothesis. Beyond the relaxation of the normality assumption, Spearman’s test is argued to be more robust to outliers than Pearson’s *r* [25]. However, in addition to sharing the same problem of Pearson’s *r* regarding its production of large coefficient values even when there is a departure from a 1∶1 relationship (Fig. 1a–c), the Spearman’s correlation has an additional peculiarity: a ρ = 1 represents a perfect monotonic relationship between variables, not necessarily a linear one (Fig. 1d–e). Therefore, even a prey adopting a quite different strategy to that predicted by the FEAR hypothesis, such as FID increasing exponentially (Fig. 1d) or logistically (Fig. 1e) with AD, would result in a maximum correlation between variables.

Pearson’s chi-square test (χ^2^) is a goodness-of-fit metric widely applied when one wants to know whether, and by how much, the observed data fit an *a priori* prediction (or a given theoretical distribution; [25], [27]–[32]). Thus, as a standardized measure of departure (distance) from an *a priori* expectation, χ^2^ assumes that both observed and expected variables are measured on the same units and scale (e.g., both in meters). Pearson’s χ^2^ is calculated as
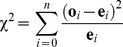
where, **o** and **e** are vectors of observed and expected values (respectively), both of length *n*. Because χ^2^ is a standardized distance between observed and expected, small values indicate small differences between observed and expected.

Tests of the FEAR hypothesis could potentially be carried out with a Pearson’s χ^2^ test. First, the FEAR hypothesis provides a clear *a priori* expectation of FID, given AD. In extreme cases, FIDs will be exactly equal to ADs (1∶1 line). Second, both AD and FID are measured in the same units (meters). However, the AD-FID relationship has an additional characteristic: AD-FID values lie in a constrained envelope [23], [33]. By definition, FID≤AD; consequently, by chance alone, FID observations with large AD are able to vary substantially more than those FIDs with smaller AD. For example, a prey that detects a predator at 10 m is able to evade it when the predator is any distance between 0 to 10 m, while a prey that detects a predator at 100 m away may choose to evade at any distance from predator between 0 to 100 m. Thus, by using χ^2^, as originally defined, the statistic would be disproportionally biased by observations with the largest ADs.

We suggest that an ideal metric to test the FEAR hypothesis must meet three criteria: (1) it must provide an intuitive measure of how close FIDs are from ADs, (2) it must be robust to outliers in order to properly capture the strategy used by most individuals observed, and (3) it must not be biased by observations with large ADs that may have a larger range of FID values.

To test the FEAR hypothesis we designed the phi (Φ) index, a distribution-free metric to evaluate the goodness of fit of a 1∶1 relationship in a constraint envelope. We initially employed simulation procedures to evaluate the statistical properties of Φ and its expectation under the null hypothesis. We then proceeded to evaluate if Φ meets the three criteria described above. We employed Φ to evaluate the FEAR hypothesis using a large empirical data set of bird species. We compared the results obtained with the conventional correlation analyses with those obtained using Φ to better understand how the choice of statistical method influences conclusions.

## Methods

### The index

The phi (Φ) index can be thought of the complement of the average standardized distance between expected (AD) and observed (FID), or, alternatively, how close the observed relationship is from the 1∶1 line:
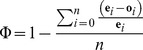



In the χ^2^ statistic the differences between observed and expected outcomes are squared, therefore equating negative and positive deviations [25]. Because AD-FID is an envelope relationship, squaring the differences is unnecessary, as e_i_−o_i_ (i.e., AD_i_−FID_i_) >0. We then divide this difference by the expected outcome (AD). By doing so we are calculating the deviance in a relative way (percent difference). This approach aims to overcome the problems of potentially excessive influential observations in cases with large ADs that result from the envelope pattern seen in AD-FID relationships. We then divide the sum of percent deviances by the sample size (*n*) to obtain a mean percent difference. Because differences (e_i_−o_i_) are standardized by their maximum value (e_i_), the mean sum of standardized differences ranges between 0 and 1. Finally, we subtract the mean percent difference from 1 so that Φ becomes a similarity index: large Φ values (Φ∼1) support the *a priori* hypothesis (no departure from 1∶1 AD-FID relationship, consistent with the FEAR hypothesis), while small Φ values (Φ∼0) show maximum departure from the expectation. We provide the R code [34] to calculate the index and test its significance in the file S1.

### Null expectation

Although Φ ranges from 0 to 1, observing extreme values of Φ must be rare. Thus, Φ requires a null model to allow comparison between an observed Φ value and its null expectation under chance alone.

To generate a null distribution of Φ we calculated the index for a series of simulated AD-FID relationships. The null models start by sampling *n* simulated AD values (sAD*_i_*) from a uniform distribution bounded between 10 and 100 m, a range usually observed in empirical studies. However, as we show in the figure S1, the results are not influenced by the choice of AD range used. Next, for each sAD*_i_*, a simulated FID (sFID*_i_*) value is sampled from a uniform distribution bounded between 0 and sAD*_i_*. Given the vectors of sAD and sFID, one simulated Φ is calculated. The process was repeated 10,000 times, generating the distribution of Φ under the assumption of independence (but constraint) between FID and AD (null expectation).

As with any frequentist null hypotheses testing, statistical significance is a balance between effect size and sample size [35]. To evaluate how hypothesis testing using Φ is influenced by a null hypothesis we varied sample size (*n*) from 4 to 200 individuals.

**Figure 2:**
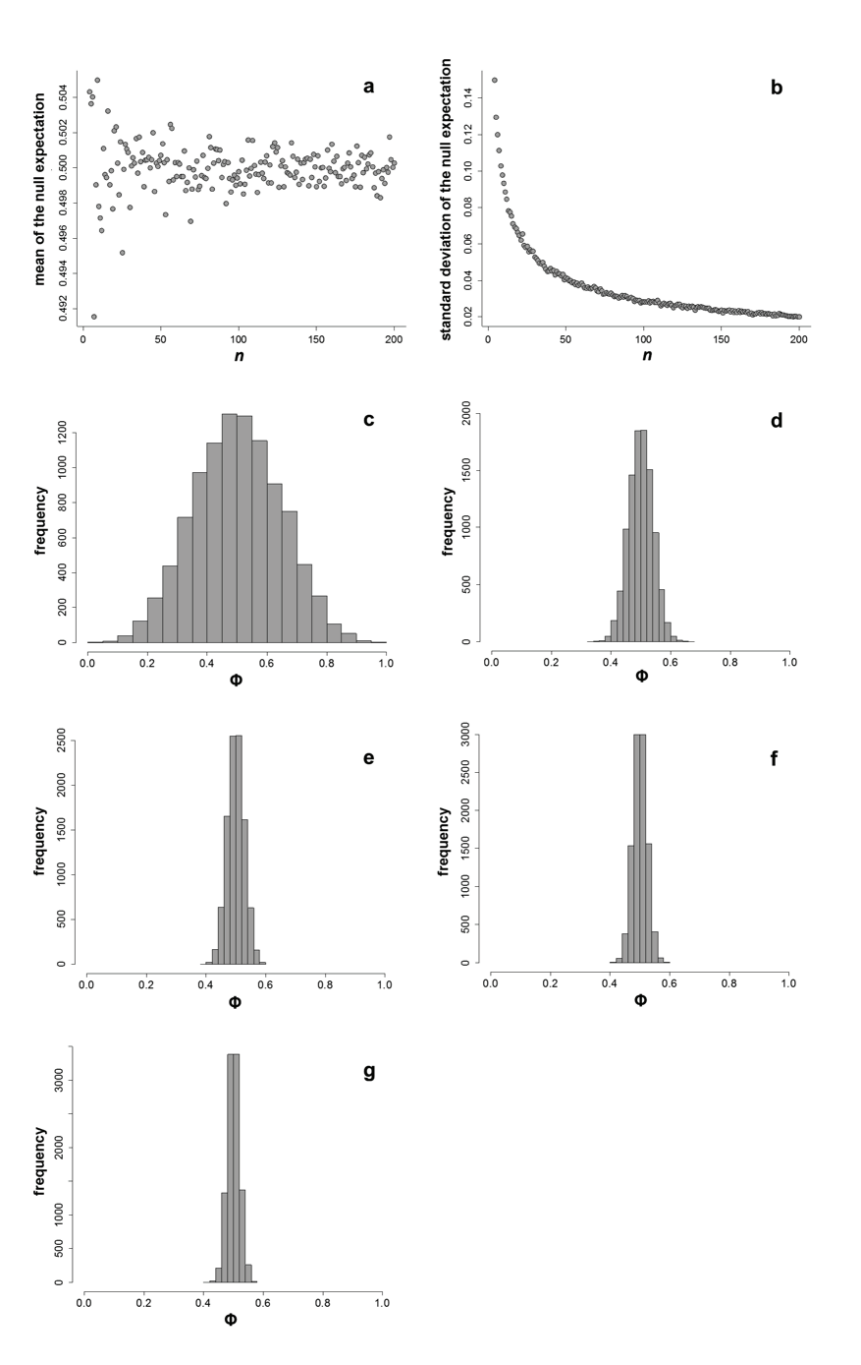
Null expectation of the phi (Φ) index using simulated data. Plots show the distribution of the expected (a) mean and (b) standard deviation of Φ as a function of sample size (*n*). Panels c–g illustrate how the null distribution of Φ becomes progressively leptokurtic as sample size increases: respectively, 4, 50, 100, 150, and 200.

The null model for Φ has a mean expectation of 0.5 (Fig. 2a), and the greater the departure of an observed Φ from 0.5, the larger the magnitude of the effect. Despite the wide range of simulated sample sizes (4 to 200), the mean of the null expectation varied narrowly (0.005 for the worst case scenario of *n* = 4). A mean expectation of 0.5 is obtained because most random FIDs generated by a uniform distribution (which represents a total absence of an *a priori* escape strategy) is homogenously spread along the envelope range (AD-0), so that, on average, the percent distance of the sFID fall in the intermediate point between flush early and flush later. The standard deviation of the null expectation shows an exponential decrease as sample size increases (Fig. 2b), indicating that the distribution of the null expectation becomes progressively leptokurtic as sample size increases (Fig. 2c–g).

However, an important point must be highlighted. Significance tests tell us whether an observed pattern is random or not, but the results of a significance test tell us nothing about whether the effect is large or small. Indeed, there is a large amount of literature arguing that one should base inferences on the effect size [35], [36]. Practically, this means that it is essential to calculate the magnitude and direction of the effect to judge its biological importance. To this end, our null model analysis reveals a desirable and informative property of Φ: as sample size increases, Φ-values that deviate from 0.5 are robust indices of a population that flushes later (<0.5) or earlier (>0.5).

### Type I error and power analysis

The type I error rate is the probability that a given statistic rejects the H_0_ when it is true [25], [37]. To estimate the rate of type I error in our proposed metric, we estimated the proportion of randomly generated datasets that produced statistically significant Φ-values. To do so, we first generated random datasets using a uniform distribution under the constraint sFID*_i_*≤sAD*_i_*. The sAD were bounded between 10 and 100 m. The Φ-value of each random dataset was calculated. Next, this “observed” Φ-value was compared against a null distribution generated from 1,000 iterations (as specified in *“Null expectation”* section). The *p*-value of this observed Ф was stored in a vector. This routine was repeated 1,000 times. The type I error rate was calculated as the proportion of the time in which the random data sets produced significant Φ (i.e., P≤0.05). Because type I error rate is affected by sample size [37], we conducted the described test simulating random data sets with sample size of 4, 25, 50, 75, 100, 150, and 200 observations.

Type II error is the probability of a significance test rejecting a false H_0_. For a given effect size, alpha, and sample size, the probability of having a type II error is β [25], [37]. Thus, 1- β is the statistical power of a given test [37]. To test the statistical power of Φ we compared both intermediate (0.75) and high (0.9) Φ-values against a null model (1,000 iterations). As with our type I error test, 1,000 random datasets were generated using a uniform distribution under the constraint that sFID*_i_*≤sAD*_i_*, where the sAD were bounded between 10 and 100 m. To simulate intermediate and high Φ-values, we simulated the sFID by multiplying the sAD vector by 0.75 and 0.9, respectively, and then calculated Φ using these vectors. The next steps were the same as those in the type I error test. We calculated the power as the proportion of the time that simulated 0.75 and 0.9 Φ-values were significant (equivalent to subtracting the number of non-significant Φ-values from 1).

Table 1 summarizes the results of both type I error and the power analysis of Φ. Regardless of the sample size used, Φ has type I error rates below the nominal rate commonly accepted (<5% at α = 0.05). Moreover, we demonstrated that Φ is a powerful test. With only one exception, all simulations had the maximum power ( = 1), because all simulations were significant in both intermediate and high Φ (Table 1). The intermediate Φ with the lowest sample size (*n* = 4) had a power of 0.915 (Table 1), considerably above the standard level of 0.8 [37]. Such findings demonstrate that Φ can be reliably applied even with small datasets.

**Table 1:** Type I error rate and statistical power of Φ with a variety of sample sizes.

N	type I error	power (0.75)	power (0.9)
4	0.042	0.915	1
25	0.049	1	1
50	0.047	1	1
75	0.045	1	1
100	0.043	1	1
150	0.048	1	1
200	0.033	1	1

Power analyses were conducted with a Φ = 0.75 and Φ = 0.9.

### Robustness to outliers

A good metric to test the FEAR hypothesis should be simultaneously robust to outliers and not biased by large departures when AD is large (due to the constraint envelope). Here we used a worked example to evaluate how Φ behaves in both cases. We compared Φ with the outcome of the Pearson’s *r*, Spearman’s ρ, and with a traditional Pearson’s χ^2^. Although Pearson’s χ^2^ is commonly applied to categorical data [28], the Pearson’s χ^2^ statistic can also be used with continuous data (as shown below) and its significance tested against a null model respecting the constraint FID≤AD. By comparing results from Pearson’s χ^2^ with Φ we aim to demonstrate how Pearson’s χ^2^ is an inadequate statistic to evaluate the FEAR hypothesis because it is disproportionately affected by deviance in large AD values.

**Figure 3:**
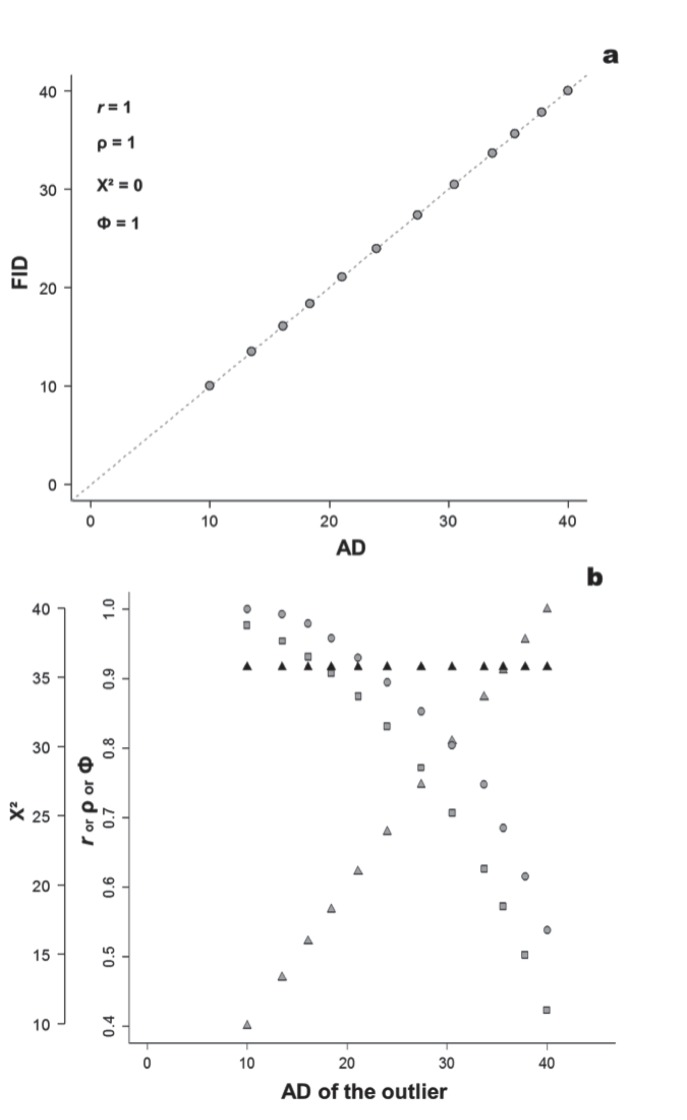
a) Relationship between alert distance (AD) and flight initiation distance (FID) of a hypothetical species that strictly followed the FEAR hypothesis (i.e., all FID = AD). Outcome of the four statistics and the 1∶1 line is shown on plot. b) The relationship between magnitude of the AD of outlier (where FID was set to 0; see text for detailed methods) and the resulting calculation of alternative metrics. Squares, Pearson’s *r*; circles, Spearman’s ρ; grey triangles, Pearson’s chi-squared (χ^2^), and black triangles, phi index (Φ).

To test the robustness to outliers, we first created a hypothetical species that rigorously follows the FEAR hypothesis (i.e., all FID = AD; Fig. 3a). To simulate the outliers, we randomly chose one of the twelve observations of the hypothetical species and reduced its FID from FID = AD to FID = 0, while keeping the remaining observations (11) unchanged. With these reduced data sets we then calculated the four metrics (Φ, *r*, ρ, χ^2^).

As we see, *r,* ρ, and χ^2^ were affected as a function of the magnitude of the AD of the outlier (Fig. 3b). As the magnitude of the outlier’s AD increases, χ^2^ increases, and *r* decreases. In contrast, because Φ standardized the differences between AD and FID (by dividing by AD), the observed Φ value remains exactly the same regardless of the magnitude of AD of the outlier. This exercise demonstrates that using relative differences between expected (AD) and observed (FID) outcomes, as opposed to natural differences used by a χ^2^ statistic, provides Φ with the necessary robustness to deviations along the range of magnitudes of AD. This is a desirable propriety of a metric aimed to test relationships when there is a constrained envelope.

Our hypothetical species example had the ADs homogeneously distributed (ca. 3 m of difference between them). It showed how conventional metrics are biased as a function of the envelope relationship, but it does not directly address the issue of influential points with significant leverage. To test how the four metrics are affected by influential observations in constrained envelope relationships, we used the same hypothetical species, but replaced the previous largest AD value (40 m) with progressively larger ADs (respectively, 50, 60, 70, and 80 m).

**Figure 4:**
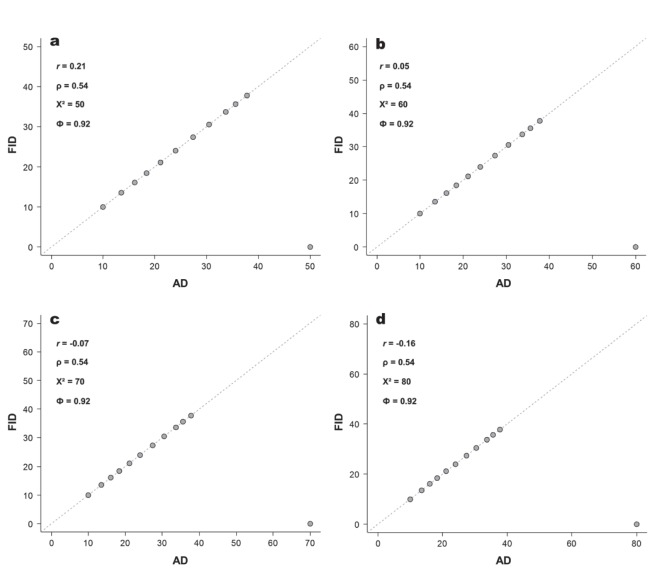
Plots of the relationship between alert distance (AD) and flight initiation distance (FID) of the hypothetical species when the largest outlier (where FID was set to 0) was changed from an AD of 40 m to, respectively, 50 m (a), 60 m (b), 70 m (c), and 80 m (d). Outcome of the four statistics (Pearson’s *r, r;* Spearman’s ρ, ρ; Pearson’s chi-squared, χ^2^; and phi index, Φ) and the 1∶1 line is shown on their respective plots.

Figure 4 illustrates the disproportionate effects a single outlier with high leverage has on *r* and χ^2^, as well as illustrates how the Φ and ρ remains unchanged. Spearman’s ρ, although substantially affected by outliers (ρ was reduced from 1 to 0.54 because a single outlier; Fig. 4), is robust to influential points. Because ρ calculates the correlation between *ranked* variables, the values of the largest observations will always be set as the first position and will yield exactly the same ρ regardless of the absolute or relative difference between the first and the second largest AD values.

## Worked Example with Empirical Data

### Data

Here we employ Φ to test the FEAR hypothesis using a large dataset with 75 bird species. These species represent most species (75 of 79 species; 95%) that were previously used in a meta-analysis which found support for the FEAR hypothesis using Pearson’s correlation coefficient [23]. Thus, the bird dataset is suitable for a comparison between statistical methodologies. The 75 species used here represent those species used in the primary studies of one of the authors (Blumstein) and were used because we had the raw data required for the analysis. In the primary studies, the effect of detection and monitoring on FID was frequently studied by using starting distance as proxy of AD (e.g., [6], [14], [38]). However, because there are some concerns about the validity of doing this [33], we extracted from the dataset only those FID observations in which the AD was recorded. By doing so, we reduced the sample size from that previously studied (more details are supplied in Tab. S1).

### Analysis

We compared the Φ outcomes with the outcomes of *r* and ρ. Because of the envelope constraint, traditional hypothesis testing cannot be carried out with the *r* and ρ values since the expected distribution differs greatly between constrained and non-constrained relationships [33] (see Fig. S2). Hence, the significance of the *r*s and ρs calculated for the 75 species were tested using the same null model described in the “null expectation” section (i.e., respecting the AD≥FID constraint).

Closely related species are more likely to have similar phenotypes because of their common ancestry, which makes data points statistically dependent [39]. We then estimated the mean *r*, ρ, and Φ for our set of 75 bird species by fitting a phylogenetic generalized least-squared model (PGLS; [40]). We ran an intercept-only model with PGLS to estimate the “phylogenetically correct mean” (*sensu* [41]). We use such a strategy because the intercept of this model is the same as the average value of the dependent variable, and the PGLS permits us to factor in the phylogenetic dependence [41]. PGLS was calculated using the “pGLS” R package [42]. We used the most recent avian phylogeny [43] (Fig. S3). But, since this phylogeny was built using Bayesian methods, we randomly selected 100 phylogenies from those available (http://birdtree.org/) and ran the analysis for each tree. Results were very similar regardless of phylogenetic tree. Thus, we conservatively used those with the least overall mean effect size.

### Results of the worked example

**Figure 5:**
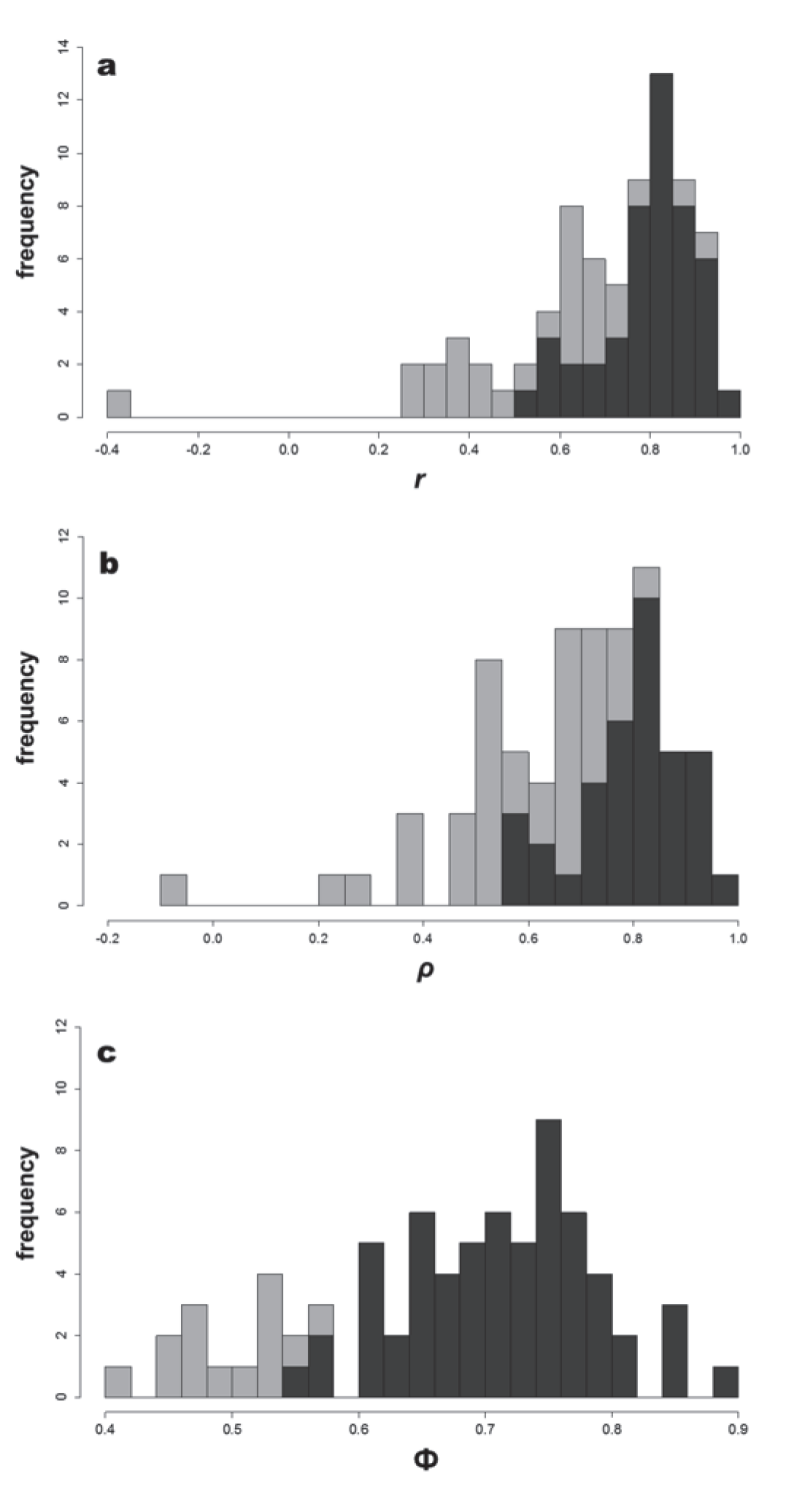
Frequency distribution of (a) Pearson’s *r*, (b) Spearman’s ρ, and (c) phi (Φ) indices (light grey bars) for the 75 avian species studied. Number of significant indices (*P*≤0.05) in each interval of values is shown by the dark grey bars.

We calculated *r,* ρ, and Φ of the AD-FID relationship for all 75 bird species (Tab. S1). Calculated *r-*values ranged from −0.35 to 0.96 with the most frequent values occurring between 0.85 and 0.9 (13 species; 17%; Fig. 5 and Tab. S1). Calculated ρ*-*values ranged from −0.06 to 0.96 with the most frequent values also occurring between 0.85 and 0.9 (11 species; 15%; Fig. 5 and Tab. S1). Calculated Φ-values ranged from 0.42 to 0.9 with the most frequent ones occurring between 0.75 and 0.8 (19 species; 25%; Fig. 5 and Tab. S1). When the FEAR hypothesis was evaluated with Pearson’s *r*, 63% (47) of the AD-FID relationships were significant, while 49% (37) relationships were significant using ρ, and 81% (61) relationships were significant using Φ (Fig. 5 and Tab. S1). Overall, the mean values were: *r = *0.75 (SE = 0.24), ρ = 0.71 (SE = 0.20), and Φ = 0.71 (SE = 0.09).

**Figure 6:**
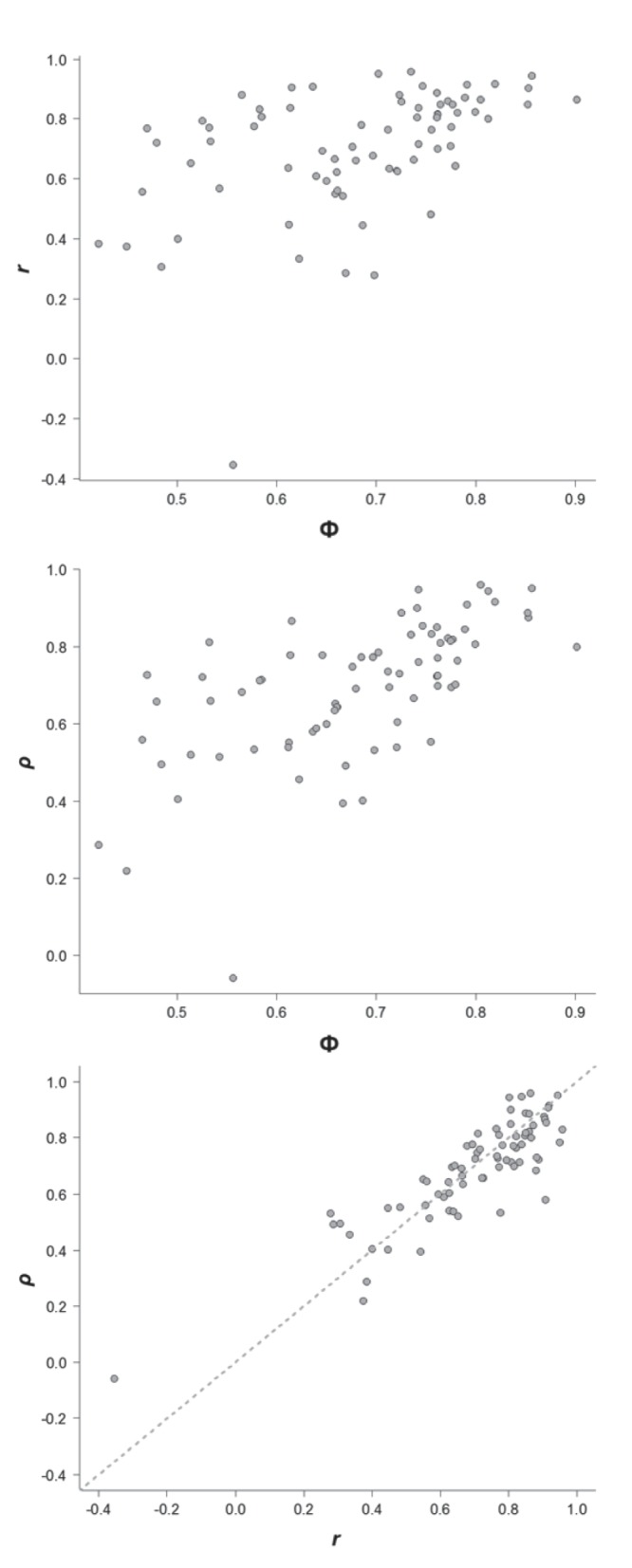
Relationship between (a) phi index (Φ) and Pearson’s *r* (*r*), (b) Φ and Spearman’s ρ (ρ), and (c) *r* and ρ for the 75 avian species studied. The dashed line in plot c represents a 1∶1 relationship.

A comparison among the metrics outputs showed a markedly heteroscedastic relationship between Φ and Pearson’s *r* and between Φ and Spearman’s ρ (Fig. 6). The variance of both *r* and ρ increased as Φ decreased (Breusch-Pagan test; *r*: χ^2^ = 15.27, d.f.  = 1, *P*<0.001; ρ: χ^2^ = 13.12, d.f. = 1, *P*<0.001). The regression analysis also showed how *r* and ρ are strongly related (intercept = 0.173, *b* = 0.737, *P*<0.001, R^2^ = 0.75). Plots 6a and 6b show that some species with a Φ around 0.5 (the mean null expectation) had large effect sizes when evaluated using correlational analysis (*r* and ρ≈0.8). However, higher Φ values (above 0.78) were associated exclusively with high *r’*s and ρ’s (Fig. 6). This result suggests the absence of outliers that bias estimates of correlation coefficients in species with high index values.

**Figure 7:**
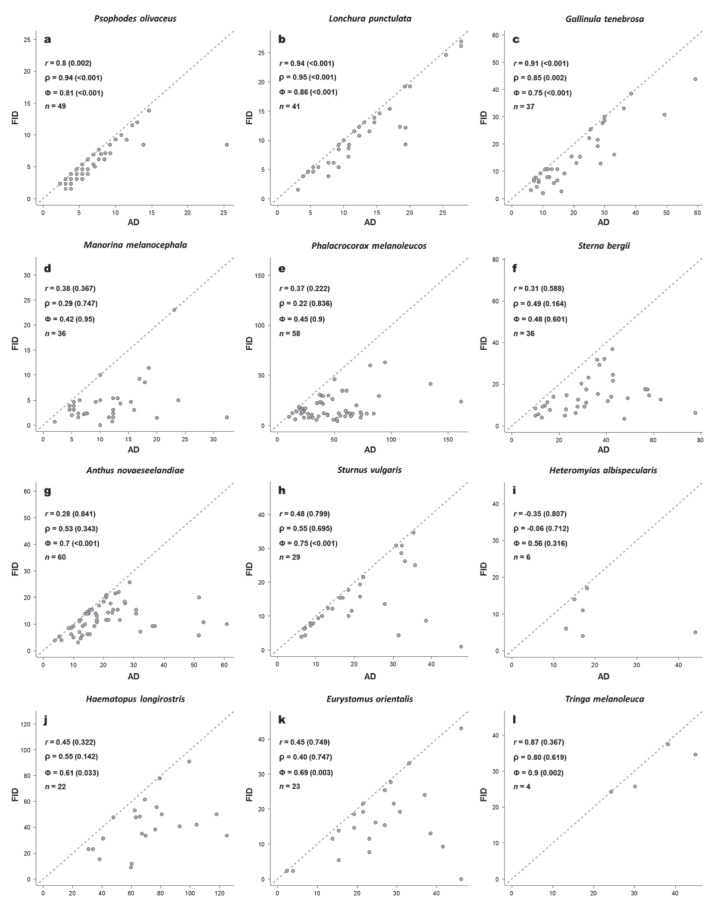
Examples of relationships between alert distance (AD) and flight initiation distance (FID) of selected avian species used in our empirical test. The FEAR hypothesis tested using both correlation coefficients (*r* and ρ) and phi (Φ) index values are shown along with their associated *P*-values (in parentheses). Plots a–f illustrate the cases in which the three metrics lead to similar conclusion, whereas plots g–l illustrate the cases where conclusions drawn from the *r* and ρ results diverge from those of Φ. *n* = sample size. Species name is indicated in the top of their respective plot. The 1∶1 relationship (dashed) is shown.

In some species, the three metrics led to similar conclusions about whether a species did or did not flush early (Fig. 7). For example, some species had both large and significant relationships regardless of the metric used (Fig. 7a–c), consistent with the FEAR hypothesis. Conversely, other species had both low and non-significant relationships using the three metrics (Fig. 7d–f), which are not consistent with the FEAR hypothesis.

Interestingly, the conclusion about whether or not a species followed the FEAR hypothesis was metric-dependent for some species. There were cases in which the effect of outliers and influential points led to an underestimation (and hence non-significance) of the correlation coefficients (Fig. 7g–i). For example, four influential points in *Anthus novaeseelandiae* (*n* = 60), and three in *Sturnus vulgaris* (*n* = 29). *Heteromyias albispecularis* (Fig. 7i) was an extreme example of high influential points creating a negative relationship between AD and FID (*r* = −0.35 and ρ = −0.06), despite all metrics being non-significant in this case. However, non-significant AD-FID relationships calculated using correlation analyses were significant when measured by Φ in other cases (Fig. 7j–l). Despite all metrics yielding relatively high values, the low sample size (*n* = 4) led *Tringa melanoleuca* to have a non-significant *r* and ρ values but a highly significant Φ-value. This finding is unsurprising because a high correlation coefficient from a small sample size is expected only by chance [26]. Conversely, as we showed here, Φ is a powerful test even when we have low sample sizes (Tab. 1).

## Discussion

In this study we proposed and evaluated a new index (phi, Φ) designed to test the Flush Early and Avoid the Rush (FEAR) hypothesis, which states that prey flee soon after they begin monitoring a potential predator that is approaching it to reduce the cost associated with on-going monitoring of the predator’s approach [21]. The FEAR hypothesis is supported by a large positive correlation between alert distance (AD) and flight initiation distance (FID). Our new index possesses the mathematical and statistical properties necessary to robustly take into account the peculiarities of the AD-FID relationship. These include the 1∶1 expectation, the envelope constraint, and expected heteroscedasticity. Additionally, we showed that Φ is a powerful test, even with low sample sizes. The Φ is accurate because the departure of the observations from the 1∶1 line is calculated as a per cent deviation. Thus, any individual observation has potentially the same weight in the final statistic (all can range from 0 to 1) regardless of the distance at which an individual is first alerted to a predator (AD). By doing so, the index does not put extra weight on the largest ADs which are a potential source of error given the envelope relationship between AD and FID.

To demonstrate the utility of Φ, we contrasted the results of calculating Φ with the traditional methods, the standard Pearson’s linear correlation coefficient and Spearman’s rank correlation. We showed that the choice of metric influences the conclusion drawn about the FEAR hypothesis. For example, by using empirical data, we detected that 30% more species behaved in a way that was consistent with the FEAR strategy when tested with Φ than when tested with Pearson’s linear correlation coefficient. We must emphasize that this was not over-attribution, because by examining individual species’ AD-FID relationships we were able to see how the presence of influential observations biased our potential conclusions that might be draw from a traditional correlational analyses.

Although we have shown that the conclusions one draws about a species following the FEAR hypothesis may be metric-dependent, the overall effect size for 75 species here is consistent with avian species generally flushing early. This conclusion is the same as that of previous meta-analysis [23] and reassures us that monitoring predators should be an important part of the economics of escape behavior. Since Φ requires raw data to be calculated, it may not be suitable for some comparative analyses where researchers only have access to mean values reported in the literature. Nevertheless, we suggest that researchers test the FEAR hypothesis in other taxa using the metric proposed here so as to evaluate the FEAR hypothesis with independent data. As a measure of effect size, Φ is an intuitive estimate of how much a species follows the FEAR hypothesis (or random anti-predator strategy, or flush later strategy). Thus, Φ can be used in future as a response variable used to investigate potential covariates predicting prey’s flight decisions.

### Limitations of alternative methods to test the FEAR hypothesis

Recently two alternative algorithms were proposed to test AD-FID relationships [33], [44]. Nonetheless, we believe that while these were valuable suggestions, and may be appropriate in some circumstances, Φ may be a preferred metric.

Chamaillé-Jammes and Blumstein [44] suggested that the FEAR hypothesis could be tested using quantile regression. Because of the envelope constraint in AD-FID relationship, data frequently show heteroscedasticity (the variance increases with AD). As a result, data may violate a critical assumption of regression analysis [25], [26], [29]. Under a quantile regression, however, heterogeneous variance is not a problem since linear relationships are modeled on quantiles of the range of response variable [45]. Nonetheless, while a valuable contribution, they noted that quantile regression requires large sample sizes to be implemented (>50; [44]). The required sample size can be a real barrier in interspecific studies where sampling effort varies, and for rare species, where it is simply difficult to obtain data. For example, only 22% of species in our data set had *n*>50. As an alternative metric, Φ has the advantage of simultaneously overcoming the problem of potentially heterogeneous variances (thus we do not need to invoke the homoscedasticity assumption) and small sample sizes, since Φ provides a robust estimate of AD-FID relationship even for small samples.

Because the AD-FID relationship is constrained by an envelope, Dumont et al. [33] noted that an spurious relationship between AD and FID should be expected. They suggested a regression-based approach that tests the significance of the observed slope against a null model. While recognizing the spurious relationship was an important advance, their method should be cautiously applied.

First, the Dumont et al. [33] method focuses on testing the significance of the slope while ignoring the intercept. However, when the intercepts change, comparisons among simulated slopes are not meaningful [26], [46]. For example, a slope of 0.6 with an intercept of 0 predicts a very different relationship than a slope of 0.6 and an intercept of 20 (as shown in Fig. 1). Moreover, because of the envelope relationship, an AD of 0 m must have an FID of 0 m. Thus, it may generally be recommended to force the intercept through the origin. Indeed, we suggest that when testing the FEAR hypothesis, it is essential to set the intercept to zero so that a slope ∼1 illustrates individuals that flush early (Fig. 1), and a slope ∼0 illustrates individuals that flush later.

Second, an assumption of the regression is that residuals follow a normal distribution [26]. While recognizing that regression estimates are robust to a weak to moderate violation of this assumption, substantial deviations reduce our confidence in parameter estimates [25], [26], [47]. For example, in our dataset, 30 species (40%) critically violated the normality of residuals assumption. For these species, the Dumont et al. [33] approach might have produced unreliable results. As we have described above, Φ is not sensitive to distributional assumptions; a feature that makes it particularly suitable for these analyses.

Finally, as with any regression analysis, the Dumont et al. [33] method must guarantee that data are well described by some model [26], [47]. For instance, Dumont et al. [33] used a model selection approach where they contrasted linear, logarithmic and polynomial regressions to seek the best model describing their data. However, the fact that a model selection approach always produces the best model(s) does not mean that they are “good” models [48]. If all competitive models are poor, the criterion will select the least poor model as the best model (this is usually the one(s) with the fewest parameters; [48]). The considerable variation (expected from data that follow a constraint envelope) means that slope estimates are particularly sensitive to outliers. Because of these points, we suggest that when using the Dumont et al. [33] method one must not rely exclusively on model selection, but rather use *r* ^2^ to both judge the importance (i.e., effect size) of the predictor(s), as well as the adequacy of the selected model [35], [36], [48].

### Final remarks

Evaluating the FEAR hypothesis is both of academic and applied interest. Academically, the FEAR hypothesis suggests that AD effect must be accounted for when studying the effects of other factors on optimal escape decisions (such as a predator’s speed or prey’s distance to refuge; [1], [2]). FEAR is also important for applied reasons. Because FID is frequently used to design set-back zones to reduce impact of humans on wildlife [10], [12], [49], an understanding what influences FID can help wildlife managers to develop more effective protected areas [11], [13]. Furthermore, because over the last decade the optimal escape literature has demonstrated that AD is probably the main predictor of FID for many species [23], we suggest it is essential to take AD into account for designing set-back zones and properly measuring the AD-FID relationship.

We have presented Φ as a way to evaluate the FEAR hypothesis. However, we suggest that Φ can also be used to test for 1∶1 expectations in other situations where the relationship between two variables is constrained by an envelope. For instance, Blumstein [50] studied factors influencing maximum running speed by regressing distance run against run time. Logically, such a regression must be forced through the origin, and logically, there can be more variation in run time as distance run increases. Thus, the Φ statistic could be potentially useful in such analyses if one expected a 1∶1 relationship between the variables. As we have shown in the present study, the use of conventional metrics can lead one to erroneous conclusions about expected 1∶1 relationships if the properties of the envelope constraint are neglected.

### Ethics Statement

This study was carried out with approval of the Macquarie University Animal Care Committee (protocol #99021) and the University of California Los Angeles Animal Research Committee (IACUC #2000-147-01). Data were collected on public and private land after acquiring required permits. By design, experimental approaches were designed to create only a brief disturbance and we are not aware of any lasting harm caused by the experimental approaches. In addition, and to reduce the likelihood of any negative effects, endangered species were not targeted, and we only targeted birds away from their nests.

## Supporting Information

Figure S1 **Null expectation of the phi (Φ) index using simulated data.** From left to right, columns shows the null distribution of simulated data with alert distance ranging from 1–1.01 m, 10–100 m, and 75–200 m. From top down, plots illustrate the distribution of the expected mean and standard deviation together, expected mean alone, and standard deviation alone of Φ as a function of sample size (*n*). Histograms illustrate how the null distribution of Φ becomes progressively leptokurtic as sample size increases: respectively, 4, 50, 100, 150, and 200. Sample sizes indicated in the top of plots.
(PDF)

Figure S2 **Example of the null expectation of Pearson’s** ***r*** **(** ***r*** **) and Spearman’s ρ (ρ) from non-constrained and constrained relationships.** The null expectations were constructed by sampling 50 simulated alert distance values (sAD*_i_*) from a uniform distribution bounded between 10 and 100 m. Next, for each sAD*_i_*, a simulated flight initiation distance (sFID*_i_*) value was sampled from a uniform distribution. For the non-constrained relationships, we permitted sFID to range from −100 to 100, while for the constrained relationships, sFID varied between 0 and sAD*_i_*. Given the vectors of sAD and sFID, we calculated both *r* and ρ. The process was repeated 10,000 times. Note how the mean expectation diverges from zero in the constrained relationships.
(PDF)

Figure S3 **Phylogenetic hypothesis of the 75 avian species included in the present study.**
(PDF)

Table S1 **Summary results of the relationship between alert distance and flight initiation distance of the 75 avian species studied.** *n*, sample size; *r*, Pearson’s correlation coefficient; P(*r*), *P*-value of the *r*; ρ, Spearman’s correlation coefficient; P(ρ), *P*-value of the ρ; Φ; the phi index; and P(Φ); associated *P*-value of the phi index.
(PDF)

File S1 **R script to calculate and test the significance of the phi index.**
(R) Click here for additional data file.
