# Supplementary material for: Phi Index: A New Metric to Test the Flush Early and Avoid the Rush Hypothesis
Source: PLoS One. 2014 Nov 18;9(11):e113134. doi: 10.1371/journal.pone.0113134 (PMC4236129; doi:10.1371/journal.pone.0113134)
Supplement: Figure S1 — Null expectation of the phi (Φ) index using simulated data. From left to right, columns shows the null distribution of simulated data with alert distance ranging from 1–1.01 m, 10–100 m, and 75–200 m. From top down, plots illustrate the distribution of the expected mean and standard deviation together, expected mean alone, and standard deviation alone of Φ as a function of sample size (n). Histograms illustrate how the null distribution of Φ becomes progressively leptokurtic as sample size increases: respectively, 4, 50, 100, 150, and 200. Sample sizes indicated in the top of plots. (PDF) [file pone.0113134.s001.pdf]

Figure S1. Null expectation of the phi (Φ) index using simulated data. From left to right, columns shows the null distribution of simulated data with alert distance ranging from 1 – 1.01 m, 10 – 100 m, and 75 – 200 m. From top down, plots illustrate the distribution of the expected mean and standard deviation together, expected mean alone, and standard deviation alone of Φ as a function of sample size (*n*). Histograms illustrate how the null distribution of Φ becomes progressively leptokurtic as sample size increases: respectively, 4, 50, 100, 150, and 200. Sample sizes indicated in the top of plots.

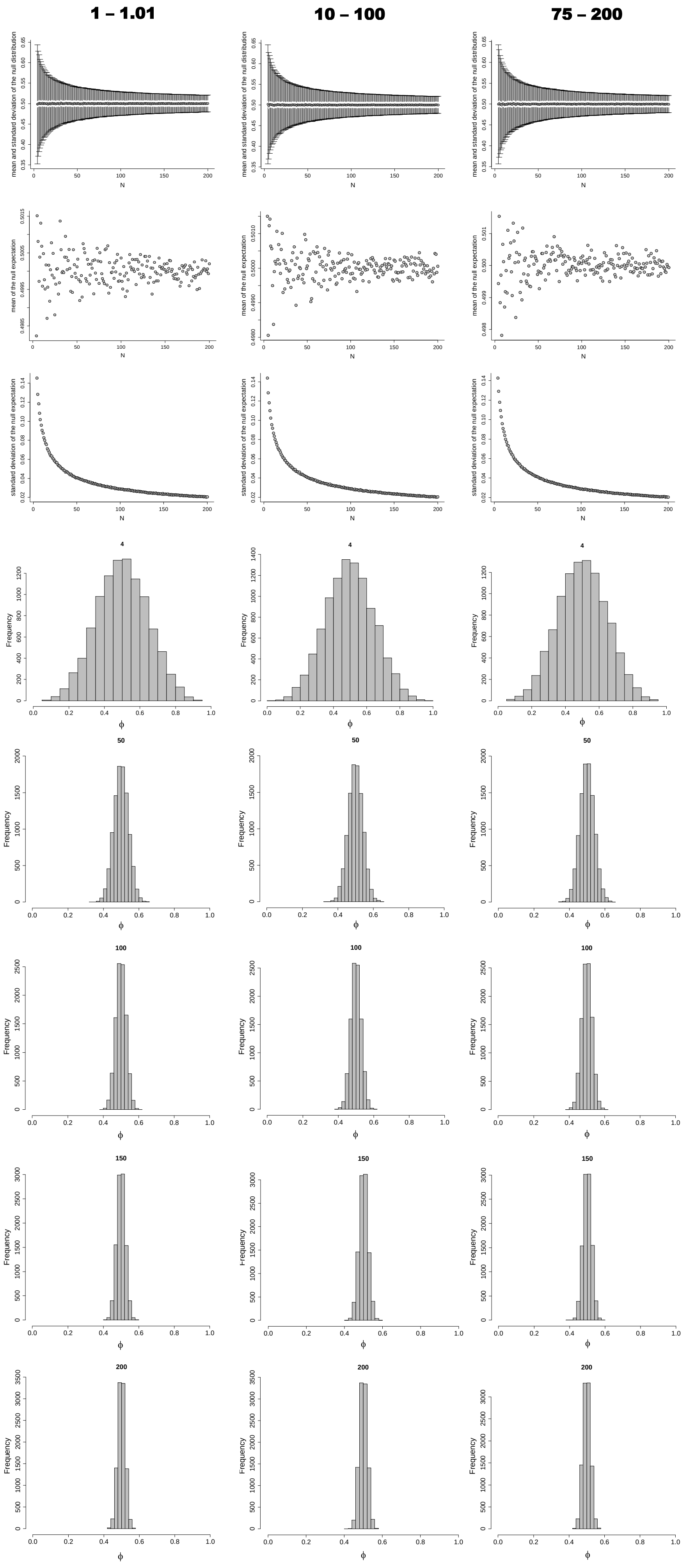
1 – 1.01
10 – 100
75 – 200
mean and standard deviation of the null distribution
mean of the null expectation
standard deviation of the null expectation
N
4
50
100
150
200
Frequency
ϕ
