# Supplementary material for: Phi Index: A New Metric to Test the Flush Early and Avoid the Rush Hypothesis
Source: PLoS One. 2014 Nov 18;9(11):e113134. doi: 10.1371/journal.pone.0113134 (PMC4236129; doi:10.1371/journal.pone.0113134)

Figure S2. Example of the null expectation of Pearson's *r* (*r*) and Spearman's ρ (ρ) from non-constrained and constrained relationships. The null expectations were constructed by sampling 50 simulated alert distance values (sAD_i) from a uniform distribution bounded between 10 and 100 m. Next, for each sAD_i, a simulated flight initiation distance ($sFID_i$) value was sampled from a uniform distribution. For the non-constrained relationships, we permitted that sFID range from -100 to 100, while for the constrained relationships sFID varied between 0 and sAD_i. Given the vectors of sAD and sFID, a *r* and ρ coefficient was calculated. The process was repeated 10,000 times. Note how the mean expectation diverges from zero in the constrained relationships.

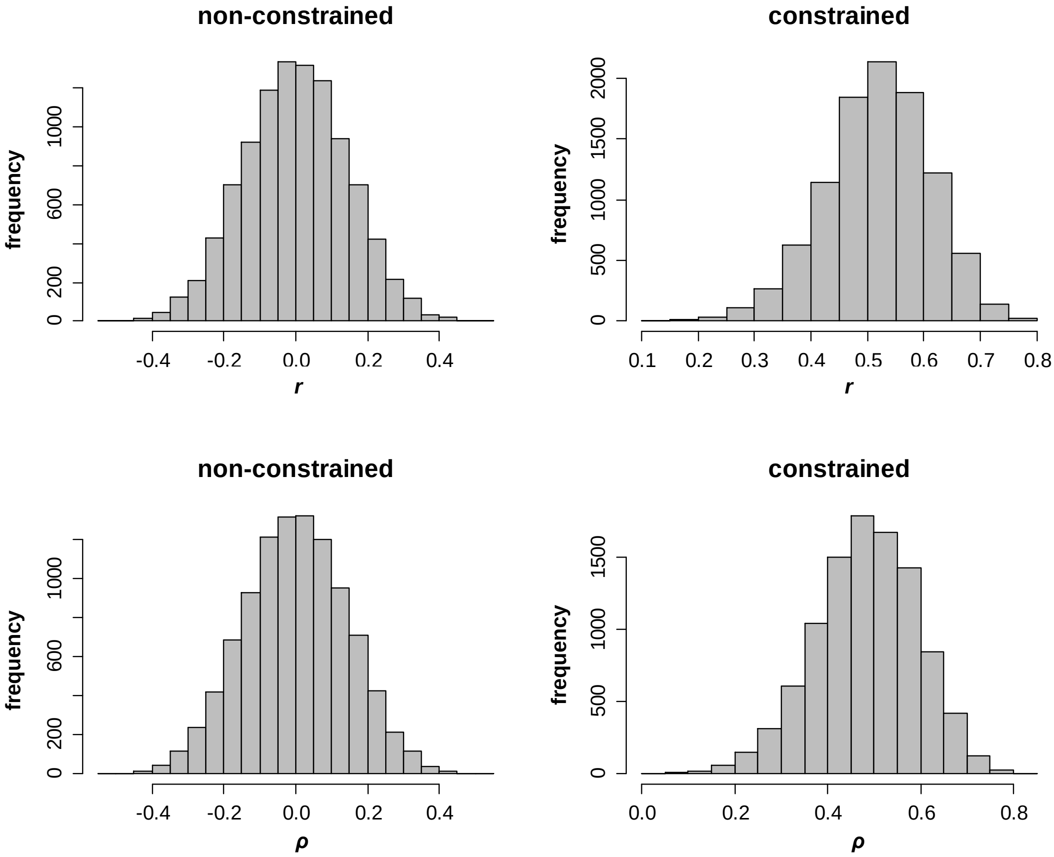

Supplement: Figure S2 — Example of the null expectation of Pearson’s r ( r ) and Spearman’s ρ (ρ) from non-constrained and constrained relationships. The null expectations were constructed by sampling 50 simulated alert distance values (sADi) from a uniform distribution bounded between 10 and 100 m. Next, for each sADi, a simulated flight initiation distance (sFIDi) value was sampled from a uniform distribution. For the non-constrained relationships, we permitted sFID to range from −100 to 100, while for the constrained relationships, sFID varied between 0 and sADi. Given the vectors of sAD and sFID, we calculated both r and ρ. The process was repeated 10,000 times. Note how the mean expectation diverges from zero in the constrained relationships. (PDF) [file pone.0113134.s002.pdf]
