# Supplementary figures and images for: Phi Index: A New Metric to Test the Flush Early and Avoid the Rush Hypothesis
Source: PLoS One. 2014 Nov 18;9(11):e113134. doi: 10.1371/journal.pone.0113134 (PMC4236129; doi:10.1371/journal.pone.0113134)

**Figure S3.** Phylogenetic hypothesis of the 75 avian species included in the present study.

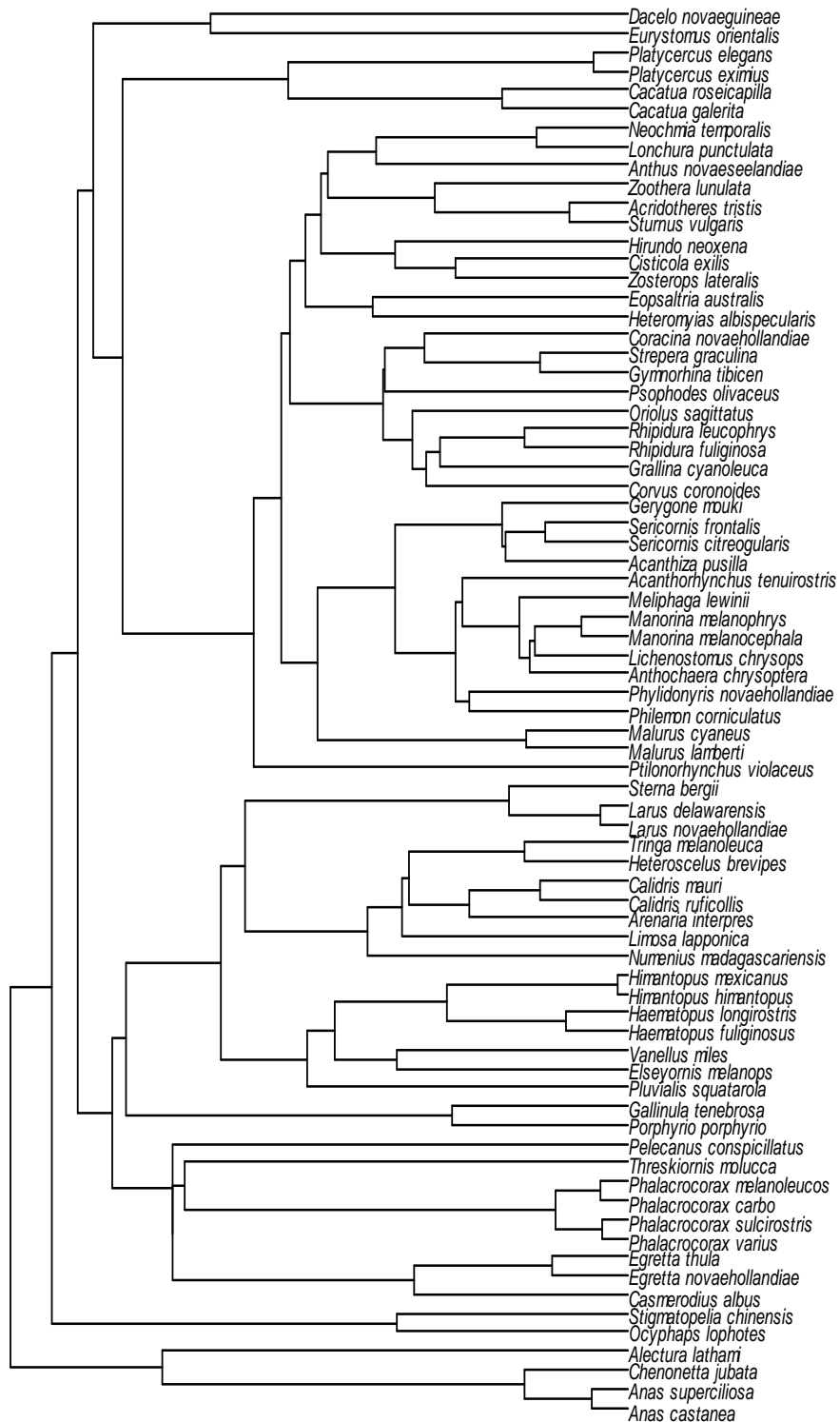

Supplement: Figure S3 — Phylogenetic hypothesis of the 75 avian species included in the present study. (PDF) [file pone.0113134.s003.pdf]
