# Supplementary material for: Phi Index: A New Metric to Test the Flush Early and Avoid the Rush Hypothesis
Source: PLoS One. 2014 Nov 18;9(11):e113134. doi: 10.1371/journal.pone.0113134 (PMC4236129; doi:10.1371/journal.pone.0113134)
Supplement: Table S1 — Summary results of the relationship between alert distance and flight initiation distance of the 75 avian species studied. n, sample size; r, Pearson’s correlation coefficient; P(r), P-value of the r; ρ, Spearman’s correlation coefficient; P(ρ), P-value of the ρ; Φ; the phi index; and P(Φ); associated P-value of the phi index. (PDF) [file pone.0113134.s004.pdf]

Table S1. Summary results of the relationship between alert distance and flight initiation distance of the 75 avian species studied. *n*, sample size; *r*, Pearson's correlation coefficient; P(*r*), *P*-value of the *r;* ρ, Spearman's correlation coefficient; P(ρ), *P*-value of the ρ; Φ; the phi index; and P(Φ); associated *P*-value of the phi index.

Species	Family	*n*	*r*	P (*r*)	ρ	*P* (ρ)	Φ	P (Φ)
Acanthiza pusilla	Pardalotidae	26	0.89	0.004	0.72	0.112	0.76	<0.001
Acanthorhynchus tenuirostris	Meliphagidae	39	0.9	<0.001	0.88	0.001	0.85	<0.001
Acridotheres tristis	Sturnidae	38	0.81	0.001	0.71	0.044	0.58	0.025
Alectura lathami	Megapodiidae	11	0.91	0.059	0.58	0.336	0.64	0.042
Anas castanea	Anatidae	55	0.85	<0.001	0.89	<0.001	0.85	<0.001
Anas superciliosa	Anatidae	50	0.87	0.002	0.84	0.012	0.79	<0.001
Anthochaera chrysoptera	Meliphagidae	39	0.66	0.069	0.69	0.054	0.68	<0.001
Anthus novaeseelandiae	Motacilidae	60	0.28	0.841	0.53	0.343	0.7	<0.001
Ardea alba	Ardeidae	30	0.91	<0.001	0.87	0.001	0.62	0.011
Arenaria interpres	Scolopacidae	46	0.61	0.017	0.59	0.050	0.64	<0.001
Cacatua galerita	Cacatuidae	39	0.78	0.007	0.53	0.460	0.58	0.049
*Cacatua roseicapila**	Cacatuidae	50	0.56	0.001	0.56	0.002	0.46	0.809
Calidris mauri	Scolopacidae	15	0.86	0.03	0.89	0.013	0.72	<0.001
Calidris ruficollis	Scolopacidae	61	0.63	0.06	0.54	0.282	0.72	<0.001
*Chenonetta jubata***	Anatidae	29	0.77	0.004	0.81	<0.001	0.53	0.101
Cisticola exilis	Sylviidae	38	0.92	<0.001	0.92	<0.001	0.82	<0.001
Coracina novaehollandiae	Campephagidae	20	0.86	0.032	0.96	<0.001	0.8	<0.001
Corvus coronoides	Corvidae	61	0.78	0.001	0.77	0.002	0.69	<0.001
Dacelo novaeguineae	Halcyonidae	52	0.88	<0.001	0.68	0.056	0.56	0.05
Egretta novaeholiandiae	Ardeidae	33	0.33	0.66	0.46	0.531	0.62	0.003
Egretta thula	Ardeidae	22	0.73	0.04	0.66	0.085	0.53	0.313
Elseyornis melanops	Charadriidae	41	0.63	0.075	0.60	0.085	0.72	<0.001
Eopsaltria australis	Petroicidae	78	0.82	<0.001	0.76	0.001	0.78	<0.001
Eurystomus orientalis	Coraciidae	23	0.45	0.749	0.40	0.747	0.69	0.003
Gallinula tenebrosa	Rallidae	37	0.91	<0.001	0.85	0.002	0.75	<0.001
Gerygone mouki	Pardalotidae	32	0.82	0.003	0.70	0.118	0.76	<0.001
Grallina cyanoleuca	Dicruridae	94	0.68	0.009	0.77	<0.001	0.7	<0.001
*Gymnorhina tibicen****	Artamidae	30	0.77	0.003	0.73	0.015	0.47	0.51
Haematopus fuliginosus	Haematopodidae	59	0.54	0.045	0.39	0.425	0.67	<0.001
Haematopus longirostris	Haematopodidae	22	0.45	0.322	0.55	0.142	0.61	0.033
Heteroscelus brevipes	Scolopacidae	46	0.55	0.073	0.65	0.022	0.66	<0.001
Heteromyias albispecularis	Petroicidae	6	-0.35	0.807	-0.06	0.712	0.56	0.316
Himantopus himantopus	Recurvirostridae	62	0.85	<0.001	0.81	<0.001	0.76	<0.001
Himantopus mexicanus	Recurvirostridae	50	0.56	0.047	0.64	0.009	0.66	<0.001
Hirundo neoxena	Hirundinidae	30	0.29	0.802	0.49	0.607	0.67	<0.001
Larus delawarensis	Laridae	12	0.72	0.098	0.66	0.197	0.48	0.603
Larus novaehollandiae	Laridae	133	0.4	0.082	0.40	0.266	0.5	0.497
Lichenostomus chrysops	Meliphagidae	28	0.96	<0.001	0.83	0.016	0.74	<0.001
Limosa lapponica	Scolopacidae	92	0.59	0.016	0.60	0.030	0.65	<0.001
Lonchura punctulata	Passeridae	41	0.94	<0.001	0.95	<0.001	0.86	<0.001
Malurus cyaneus	Maluridae	87	0.67	0.008	0.67	0.030	0.74	<0.001
Malurus lamberti	Maluridae	38	0.86	0.005	0.82	0.004	0.77	<0.001

Manorina melanocephala	Meliphagidae	36	0.38	0.367	0.29	0.747	0.42	0.95
Manorina melanophrys	Meliphagidae	44	0.84	0.005	0.95	<0.001	0.74	<0.001
Meliphaga lewinii	Meliphagidae	30	0.81	0.046	0.77	0.144	0.76	<0.001
Neochmia temporalis	Passeridae	51	0.82	0.001	0.81	0.003	0.8	<0.001
Numenius madagascariensis	Scolopacidae	42	0.81	<0.001	0.85	0.001	0.76	<0.001
Ocyphaps lophotes	Columbidae	29	0.83	0.002	0.71	0.051	0.58	0.066
Oriolus sagittatus	Oriolidae	31	0.81	0.024	0.90	0.002	0.74	<0.001
Pelecanus conspicilatus	Pelecanidae	38	0.79	0.001	0.72	0.025	0.53	0.289
Phalacrocorax carbo	Phalacrocoracidae	33	0.84	<0.001	0.78	0.007	0.61	0.015
Phalacrocorax melanoleucos	Phalacrocoracidae	58	0.37	0.222	0.22	0.836	0.45	0.9
Phalacrocorax sulcirostris	Phalacrocoracidae	36	0.65	0.016	0.52	0.177	0.51	0.394
Phalacrocorax variius	Phalacrocoracidae	25	0.57	0.067	0.51	0.187	0.54	0.242
Philemon corniculatus	Meliphagidae	52	0.71	0.015	0.75	0.007	0.68	<0.001
Phylidonyris novaehollandidae	Meliphagidae	47	0.85	<0.001	0.82	0.005	0.78	<0.001
*Platycercus elegans*****	Psitacidae	41	0.64	0.054	0.54	0.266	0.61	0.004
Platycercus eximius	Psittacidae	26	0.77	0.064	0.74	0.146	0.71	<0.001
Pluvialis squatarola	Charadriidae	25	0.77	0.02	0.70	0.124	0.78	<0.001
Porphyrio porphyrio	Rallidae	68	0.76	0.006	0.83	0.001	0.76	<0.001
Psophodes olivaceus	Cinclosomatidae	49	0.8	0.002	0.94	<0.001	0.81	<0.001
Ptilonorhynchus violaceus	Ptilonorhynchidae	20	0.71	0.238	0.82	0.051	0.77	<0.001
Rhipidura fuliginosa	Dicruridae	35	0.63	0.147	0.70	0.111	0.71	<0.001
Rhipidura leucophrys	Dicruridae	44	0.72	0.025	0.76	0.013	0.74	<0.001
Sericornis citreogularis	Pardalotidae	48	0.92	<0.001	0.91	<0.001	0.79	<0.001
Sericornis frontalis	Pardalotidae	40	0.7	0.073	0.72	0.104	0.76	<0.001
Sterna bergii	Laridae	36	0.31	0.588	0.49	0.164	0.48	0.601
Strepera graculina	Artamidae	25	0.95	0.002	0.78	0.073	0.7	<0.001
Streptopelia chinensis	Columbidae	52	0.62	0.188	0.64	0.241	0.66	<0.001
Sturnus vulgaris	Sturnidae	29	0.48	0.799	0.55	0.695	0.75	<0.001
Threskiornis molucca	Threskiornithidae	48	0.67	0.065	0.64	0.286	0.66	<0.001
Tringa melanoleuca	Scolopacidae	4	0.87	0.367	0.80	0.619	0.9	0.002
Vanellus miles	Charadriidae	37	0.69	0.087	0.78	0.013	0.65	<0.001
Zoothera lunulata	Muscicapidae	31	0.64	0.16	0.70	0.116	0.78	<0.001
Zosterops lateralis	Zosteropidae	33	0.88	0.002	0.73	0.138	0.72	<0.001

As stated in the "*Worked example with empirical data*" section in the main text, most correlation coefficients (*r*) presented here differ from those presented in Table S1 of [1] because here we used only the subset of the original data set in which FID was recorded along with AD (rather than its proxy, SD). By doing so, we reduced the sample size of some species because AD-values were not always recorded.

Despite this, four species for which the *r*-values were estimated from AD-FID data in the meta-analysis differ from that presented here. This is justified because in meta-analysis we estimated *r*-values based on statistical results provided by primary studies. Such estimations are expected to differ a bit from *r*-values calculated directly from the raw data because conversions between measures of effect sizes are not perfect [2,3]. Below we list those species that varied between this study and [1] and the associated statistic used to estimate their effect size in the meta-analysis.

* *Cacatua roseicapila*: *b*=0.442 (CI = 0.388 – 0.496).

** *Chenonetta jubata*: partial η^2 = 0.886.

*** *Gymnorhina tibicen*: partial η^2 = 0.826. Additionally, the correct sample size is 30, not 28 as inadvertently reported in the meta-analysis.

**** *Platycercus elegans*: partial r^2 of the model (= 0.493).

Supplementary References

1. Samia DSM, Nomura F, Blumstein DT (2013) Do animals generally flush early and avoid the rush? A meta-analysis. Biol Lett 9: 20130016.

2. Nakagawa S, Cuthill IC (2007) Effect size, confidence interval and statistical significance: a practical guide for biologists. Biol Rev Camb Philos Soc 82: 591–605.

3. Borenstein M, Hedges L V., Higgins JPT, Rothstein HR (2009) Introduction to Meta-Analysis. Chichester: John Wiley & Sons, Ltd. 421 p.
